# Short-windedness Would Weaken Effective Climate Policy

**DOI:** 10.1007/s10640-020-00447-8

**Published:** 2020-07-10

**Authors:** Wilfried Rickels, Sonja Peterson

**Affiliations:** Kiel Institute for the World Economy, Kiellinie 66, 24150 Kiel, Germany

**Keywords:** Climate policy, Carbon prices, Economic recovery, Corona virus, Lock-down

## Abstract

Most states have implemented quite strict measures designed to slow down the spread of the coronavirus among their populations. For most sectors, these measures have resulted in a significant reduction of economic activity, output, and hence also output-related emissions. Commitment to these measures, apparently regardless of the economic costs involved, is considered by some people to be a blueprint for the commitment required to mitigate climate change and to achieve the Paris climate targets. However, when it comes to devising an efficient climate policy, the differences between the two crises—cororonavirus and climate change—need to be taken more seriously than the similarities. Alarming have been the various calls to put a quick end to corona prevention measures and the restrictions they place on public and economic activity, indicative as they are of the priority accorded to high discount rates and the absence of precautionary thinking among policy-makers. Both the differences between the two crises themselves and the similarities in the reluctance to focus on achieving (more) long-term benefits emphasize once again the need for long-term commitment to climate policies in line with agreed targets.

Committing to rigid shutdown measures to contain the spreading of the corona virus has been undertaken on the tacit assumption that these measures will be temporary and can be loosened when the Covid-19 infection rates decrease and discontinued altogether once vaccines are available. Mitigating climate change and achieving ambitious temperature targets as set out in the Paris Agreement requires a long-term structural change taking us away from our current carbon-intensive economy to a zero-carbon and then net-negative carbon economy. As current research holds out little hope that a “perfect” vaccine in the form of solar climate engineering will be available in the future, the measures and efforts required must translate into a permanent, ongoing form of commitment. While progressive climate change and the spread of the coronavirus operate on very different time scales, impatience about the duration of corona lockdown has indicated once more a fundamental problem for (long-term) environmental concerns. Clearly, the economic and social costs associated with the emergence of the virus and the shutdown are significant (Helm 2020; OECD 2020). But any serious cost–benefit analysis would need to take into account not only the fact that different degrees of lockdown are available but also that the overall cost is affected by the expectations of agents regarding possible future re-lockdowns due to insufficient containment of the virus. Seen thus, it is anything but clear at which point in time the actual cost of lockdown would have exceeded the economic cost of the virus spreading in an unmitigated (or insufficiently mitigated) way.

During the course of lock-down measures, voices calling for a “green” recovery stimulus package centering around low-carbon investments in the aftermath of the corona crisis have make themselves heard. By contrast, advocates of postponing climate mitigation-related taxes, levies, and regulations have also entered the fray, claiming that timely recovery should not be jeopardized by any additional economic burdens. The debate on the relation between (economic) recovery and climate policies has been conducted from three major perspectives. The first of these is largely notable for general statements of intent recommending that the recovery should be “green” and sustainable, that EU climate targets should be supported, and that other environmental targets (maintaining biodiversity, etc.) need to be taken into account when designing recovery measures. Such well-meant counsels as the statement issued by the German National Academy of Sciences Leopoldina (2020) are useful in reminding us that recovery from the corona crisis should not come at the expense of neglecting other objectives and that climate policy should not be back-burnered, as was the case after the financial crisis in 2009. Otherwise they are of little practical value.

The second approach has involved rather detailed proposals calling either for a “greening” of recovery by foregrounding measures to support renewable energies, public transport, energy efficiency etc. or for a “blackening” of recovery by postponing and/or abandoning climate measures and environmental regulations. Predominantly, these proposals are representing the positions of the various interest groups involved. For example, representatives of the aviation industry try to prevent the harmonization of carbon prices on fuels with respect to kerosene and argue against the introduction of kerosene taxes. This idea resurfaces in the discussion on recovery measures by, say, the Austrian Aviation Association (2020). On the other hand, in its comprehensive list of (recovery) demands, the NGO German Environment Action (2020) urges for example for the abandonment of blue hydrogen projects (though not explaining why this is likely to stimulate economic recovery). Various other interest groups are in favor of postponing, suspending, or even abandoning existing environmental and climate regulations. For example, Janusz Kowlaski, the Polish Deputy Minister of State Assets urges “…[that] the ETS [European Emissions Trading Scheme] should be removed from January 1, 2021 or at least Poland should be excluded from the system.”^1^ Clearly, there is no point in discussing nonsensical ideas of this kind. But some of these proposals also make sensible suggestions like adjusting the German cap on renewable energy installations or abandoning the EU average fleet-consumption regulation because the former contradicts German renewable-energy targets and the latter is an inefficient instrument for regulating vehicle emissions. However, these suggestions do nothing to provide stimulus for a quick recovery. While specific processes and regulation timelines for regulations may need to be adjusted in the context of the corona crisis, sensible measures of this kind should be discussed and decided upon in the regular political process. Confining potential stimulus and recovery measures to their proper purpose does not mean imposing a ban on meaningful (climate or environmental) policies that are not associated with the corona crisis.

The third and most sensible perspective replaces specific proposals with (sustainability) assessment guidelines like those suggested by the World Bank (2020). While hardly any possible recovery measure would perform well against the comprehensive list of criteria provided by the World Bank, such guidelines are helpful in arguing against interest group driven proposals. The World Bank has suggested that potential measures up for consideration as part of a recovery strategy need to be assessed against both, short- and long-term criteria, an example for the former being the expected economic multiplier associated with certain measures. Bayer et al. (2020) suggest that income transfers (as planned under the US CARES package) perform well against this specific short-term criteria: they could help to stabilize private-sector spending and the multiplier could increase to 2 in the case of transfers being conditional—but not related to emissions but to the propensity to consume, i.e. conditional on being unemployed. However, private-sector spending like this should not imply any unintended adverse effects on essential long-term structural change that might arise from such things as (temporarily) adjusted risk preferences. Once postponed or stimulated demand and investment take place during the recovery process, carbon-price signals are vital in providing technology-neutral incentives for low-CO_2_ purchasing and production decisions.

Overloading stimulus or recovery packages with too many (emission-related) conditions performs poorly against the short-term criteria with respect to a timely recovery. Even worse, the inclusion in recovery packages of various detailed suggestions from the various interest groups usually results in a non-transparent, rent-seeking, and political bargaining process in which it remains unclear whether (sensible) individual emission-related decisions are being prioritized at the expense of a more challenging long-term climate policy. Accordingly, accounting for the long-term criteria requires that existing or planned climate policies providing incentives for emission reductions and technological innovation should remain in place and not be postponed, let alone weakened. Otherwise, uncertain (short-term) recovery impulses most likely come at the cost of less efficient emission-reduction paths in the long term.

## Footnotes

1 https://www.reuters.com/article/us-health-coronavirus-poland-ets/eu-should-scrap-emissions-trading-scheme-polish-official-says-idUSKBN2141RC.
